# Virulence and Antibiotic Resistance Characteristics of *Vibrio* Isolates From Rustic Environmental Freshwaters

**DOI:** 10.3389/fcimb.2021.732001

**Published:** 2021-08-19

**Authors:** Oyama Gxalo, Tennison O. Digban, Bright E. Igere, Ola A. Olapade, Anthony I. Okoh, Uchechukwu U. Nwodo

**Affiliations:** ^1^South Africa Medical Research Center (SAMRC) Microbial Water Quality Monitoring Centre, University of Fort Hare, Alice, South Africa; ^2^Applied and Environmental Microbiology Research Group (AEMREG), Department of Biochemistry and Microbiology, University of Fort Hare, Alice, South Africa; ^3^Biology Department, Albion College, Albion, MI, United States

**Keywords:** antibiotic resistance, *Vibrio* isolates, virulence genes, MARI, South Africa

## Abstract

The study investigated the occurrence of antimicrobial resistance genes and virulence determinants in *Vibrio* species recovered from different freshwater sheds in rustic milieu. A total of 118 *Vibrio* isolates comprising *Vibrio fluvialis* (n=41), *Vibrio mimicus* (n=40) and *V. vulnificus* (n=37) was identified by amplification of *ToxR*, *vmh* and *hsp60* genes. The amplification of virulence genes indicated that *V*. *mimicus* (*toxR*, *zot*, *ctx*, *VPI*, and *ompU*) genes were detected in 12.5%, 32.5%, 45%, 37.5% and 10% respectively. *V*. *fluvialis* genes (*stn*, *hupO* and *vfh*) were harboured in 48.8%, 14.6% and 19.5% isolates congruently. The other virulence genes that include *vcgC* and *vcgE* were observed in 63.1% and 29% of isolates belonging to *V*. *vulnificus*. With the exceptions of imipenem, meropenem and ciprofloxacin, most isolates exhibited more than 50% resistance to antibiotics. The antimicrobial resistance was more prevalent for polymyxin B (100%), azithromycin (100%) and least in ciprofloxacin (16.1%). Multiple antibiotic resistance index range was 0.3 and 0.8 with most isolates showing MARI of 0.8. The *bla*TEM, AmpC, *bla*GES, *bla*IMP, *bla*OXA-48 and *bla*KPC genes were detected in 53.3%, 42%, 29.6%, 16.6%, 15%, 11.3% and 5.6% of the isolates. Non-beta lactamases such as streptomycin resistance (*aadA* and *strA*), gentamicin resistance (*aphA1*) and quinolone resistance gene (*qnrVC*) were found in 5.2%, 44.3%, 26% and 2.8%. Chloramphenicol resistance genes (*cmlA1* and *catII*) were found in 5.2% and 44.3% among the isolates. Our findings reveal the presence of antimicrobial resistance genes and virulent *Vibrio* species in aquatic environment which can have potential risk to human and animal’s health.

## Introduction

Water containing infectious pathogens has been associated with ailments globally amongst human populations (Ramírez-Castillo et al., 2015). The importance and need for better water quality cannot be over-emphasized. Water scarcity is becoming more serious due to urbanization and population growth (Tzanakakis et al., 2020) hence there is an increase in demand for water. However, in most countries a strategy to minimize water shortage has been put in place which involves treating and recycling wastewater (Voulvoulis, 2018). Many treatment plants for wastewater in South Africa discharge their final effluents openly into adjoining streams or rivers used by the nearby villages/suburb for their several domestic needs (Wall, 2018). However, reports suggest that some wastewater are not properly treated and therefore discharge effluent of poor quality (Edokpayi et al., 2015). Wastewater and sewage from several sources contribute to spreading antimicrobial resistance (AMR) to the environment and adversely impacting on the habitation of aquatic animals and their surrounding water (Fouz et al., 2020). Several studies have emphasized on the effect of sewage as a huge environmental pool of AMR, as it signifies a suitable milieu for AMR organisms and antimicrobial resistance genes to thrive (Rizzo et al., 2013: Singer et al., 2016). Numerous resistant pathogens have been identified in various environmental sewage, hospital effluents, surface water and wastewater in treatment plants (Lundborg and Tamhankar, 2017). The aquatic environment has become reservoir of many microbes including *Vibrios* that are potentially infectious to humans. *Vibrio* genus is grouped in the family *Vibrionaceae*, which comprises of opportunistic pathogenic microbes infecting both humans and animals (Takemura et al., 2014). *Vibrio* species are a group of Gram-negative, curved-rod shaped bacteria being regular constituent of estuarine, freshwater and marine environs (Baker-Austin et al., 2018). Their genomes are separated inside two chromosomes having been shaped by recombination and the acquiring of genes by horizontal gene transfer from other bacteria (Oliveira et al., 2017). Infections caused by pathogenic *Vibrio* remain severe public health significance. Cholera causing *Vibrio* (*Vibrio cholerae*) is implicated in serious diarrhoeal infection which can be rapidly fatal when not treated and is typically disseminated through person-to-person contact as well as infected water (Das et al., 2020). Non-cholera causing *Vibrio* species (including *V. parahaemolyticus*, *V. anguillarum*, *V. metchnikovii*, *Vibrio alginolyticus*, *V. harveyi*, *V. mimicus*, *V. fluvialis*, and *V. vulnificus*) are known to cause vibriosis that is usually contracted *via* sea water or through ingesting raw or parboiled infected seafood causing necrotizing fasciitis, self-limiting gastroenteritis, severe septicaemia (Gennari et al., 2012). *Vibrio* species pathogenic to humans produce a variety of virulence genes which contribute to some ailments (Álvarez-Contreras et al., 2021). Some of the virulence trait includes the secretion of extracellular products (ECP), thermostable direct haemolysin (*tdh*), and tdh-related haemolysin expressed in *V. parahaemolyticus*, expression of capsular polysaccharides, cytotoxicity, biofilm formation, cholera toxin and the toxin-coregulated pilus (*tcp*) in *Vibrio cholerae* (Binesse et al., 2008). The *V. fluvialis* protease gene (*vfp*), heme utilization protein gene (*hupO*) and extracellular haemolysin gene (*vfh*), are also among the *Vibrio* virulence genes (Ramamurthy et al., 2014). The emergence of *Vibrio* strains in aquatic environments have created more apprehension leading to upsurge in resistance among *Vibrio* isolates against most clinically prescribed antibiotics and this has initiated apprehension in the healthcare sector thwarting current therapeutic outcomes (Banerjee and Farber, 2018). *Vibrio* species are documented to be very sensitive to many antimicrobials that are clinically recommended for therapeutic purpose and comprises of tetracycline, cephalosporins quinolones, aminoglycosides, and folate pathway inhibitors (Elmahdi et al., 2016). The incessant abuse of antibiotics has over time contributed immensely to the rapid emergence of drug resistance microbial strains including several *Vibrio* species (Manyi-Loh et al., 2018). Antibiotics are broadly used to manage or treat bacterial ailments in humans as well as in veterinary medicine. The beginning of this millennium signifies the end of the golden era of antibiotics and the commencement of more alertness of the public health alertness on the distribution of antimicrobial resistance. Documented reports have emphasized the significant importance of various aquatic environments in the spread of AMR (Baquero et al., 2008) especially predominantly in wastewater effluents which are regarded as hot spots for horizontal genes transfer (Bouki et al., 2013). South Africa is situated in a semi-dry area and typically experience limited annual rainfalls giving rise to scarcity of portable water. Consequently, most inhabitants in the rural areas resort to the use of untreated water from vulnerable sources, like rivers, and dams for their daily supply (Fobosi, 2013). Over the years, many researchers have focused on the severity of diseases caused by *Vibrio cholerae* opting out relatively other *Vibrio* species of medical interest, some of which are emerging pathogens that are able to cause mild to severe human diseases (Osunla and Okoh, 2017). Therefore, the aim of this study was to assess the antibiogram, virulence and resistance genes of non-cholera causing *Vibrio* species recovered from aquatic sources.

## Materials and Methods

### Study Site

The Eastern Cape is one of the nine provinces within South Africa; it is separated into two Buffalo City and Nelson Mandela Bay and six district municipalities for local government purposes. The district municipalities are in turn split into thirty-one local municipalities. The Eastern Cape mostly consists of rural settlements with minimal basic services. This study was carried out in two of the six district municipalities of this region, specifically at the Amathole District and Chris Hani District Municipalities. The Chris Hani District Municipality is located in the central of the Eastern Cape hinterland, between the Eastern Cape coastline and the Drakensberg mountains and spans an estimate of 37 294 km^2^. The district municipality is located on the watershed of four river viz. Orange River, Great Fish River, Mbashe River and Great Kei River. Surface water sources supply water for most of the towns in the area while only a few depend on groundwater supplies. The Amathole District Municipality is located in the central part of the Eastern Cape and spans along the Sunshine Coast from the Fish River Mouth and along the Eastern Seaboard to the south of Hole in the Wall alongside the Wild Coast. It is also bordered to the north by the Amathole Mountain Range. The rivers examined in this study were selected due to the fact that they serve as final receiving bodies of water for the release of treated domestic sewage operated in activated sludge systems. The targeted hospitals were also chosen because they serve as referral hospitals for the indigenous inhabitants with various medical conditions, they do not have independent wastewater treatment mechanism but are joined with that of the municipal waste treatment system.

### Collection of Samples

A total of seventy-two (72) wastewater samples were collected aseptically fortnightly from the 9 sampling sites (shown in **Table 1**) over a period of 4 months (July 2018-October 2018) with sterile 500 mL Borosilicate Glass bottles. The samples were conveyed on ice in a cooler box from the sampling site to the Applied and Environmental Microbiology Research Group (AEMREG) laboratory at the University of Fort Hare for analysis within few hours of collection.

**Table 1:** Sampling sites and their coordinates.

District municipality	Sampling site	Geographical coordinates
Chris Hani district municipality	Komani River	31°54′548″S
		26°50′715″E
	QT WWTP	31°53′38.9″S
		26°53′26.61″E
	Frontier Hospital	31°53′21″S
		26°52′17″E
	Cofimvaba River	32°00′855″S
		27°35′597″E
	Cofimvaba Hospital	32°00′43″S
		27°34′59″E
Amathole District Municipality	Victoria hospital	32°46′37″S
		26°50′46″E
	Thyume River	32°41′5″S
		26°54′18″E
	Seymour upstream, downstream and final effluent	32°34′8″S
		26°45′2″E
	Fort Beaufort final effluent and downstream	32°46′44″S
		26°38′7″E

### Isolation and Identification of Presumptive *Vibrio* Species

Standard membrane procedure was employed in processing the wastewater samples (APHA, A. 1998). Each wastewater sample was serially diluted and a 100 ml volume was filtered through 0.45 μm mixed cellulose ester membrane filters (Millipore, Ireland) using a vacuum pump. The membrane filter paper was transferred onto alkaline peptone water as means of enrichment and incubated aerobically at 37°C for 18-24 h. A loopful from the alkaline peptone water was sub-cultured onto thiosulphate citrate bile salts sucrose (TCBS, Darmstadt, Germany) and incubated for another 24 h at 37°C. Four to six colonies were randomly chosen from each plate and consequently purified in fresh TCBS agar plates. Purified isolates were subcultured on Glycerol stocks (50%) and stored at -80 °C sequel to further analysis.

### Genomic DNA Extraction

Nucleic acids (DNA) from presumptive *Vibrio* species were extracted following the method described by Mapipa et al. (2021) with minor modification. The colonies were inoculated in Luria Bertani (LB) broth and incubated at 37°C for 18-24 h. The overnight broths were centrifuged (11,000 rpm for 1 min) to pellet the cells, washed with normal saline (0.85%) and re-suspended in 0.5 ml distilled water. The emulsified pellet suspension was heated at 100°C for 15 min in a heating block to release the DNA. The lysate was centrifuged at 13000 rpm for 5 min to eliminate cell debris and the supernatant was stored (−20°C) until further use.

### Presumptive *Vibrio* Isolates Confirmation

The presumptive confirmation of the *Vibrio* isolates was achieved with PCR assay using specific primers targeting a variable portion with the 16SrRNA gene. The confirmed isolates of the *Vibrio* isolates were also delineated into *V. mimicus*, *V. fluvialis* and *V. Vulnificus.* The PCR cocktail comprised of 12 μl of Master-mix (inqaba), 1μl of both forward and reverse oligonucleotide primers (Inqaba, SA), 6 μl of nuclease free water and 5μl of DNA template which summed up to 25 μl reaction mixture. The amplicons were differentiated by electrophoresis in 0.8% agarose gels containing 0.5 μg/mL ethidium bromide (Sigma-Aldrich, St. Louis, USA) run at 90 V for 50 min and visualised under a UV transilluminator (Alliance 4.7 Uvitec, Cambridge). The targeted genes, primer sequences, band sizes and cycling condition of the *Vibrio* species are shown on **Appendix 1** (**Supplementary Material**).

### Antimicrobial Sensitivity Test Performed on the *Vibrio* Isolates

Antibiotic sensitivity test was carried out using Mueller-Hinton agar (Basingshike, Hampshire, England) by the standard disk diffusion technique (Kirby-Bauer test) as endorsed by the Clinical and Laboratory Standards Institute (Clinical and Laboratory Standards Institute, 2018). Few colonies of confirmed isolates of the *Vibrio* species were reconstituted on sterile normal saline to give an approximately 0.5 McFarland standard. A lawn of the bacterial suspension was made on Mueller-Hinton agar and allowed to stand for 15 min. A panel of 12 antibiotics which are usually recommended for treating *Vibrio* infections were selected and impregnated unto the agar plates. The antibiotic disks include: ampicillin (10 μg), ampicillin-sulbactam (10/10 μg), imipenem (10 μg), meropenem (10 μg), kanamycin (30 μg), ciprofloxacin (5 μg), ofloxacin (5 μg), cefuroxime (30 μg), polymyxin B (300 μg), azithromycin (15 μg), nitrofurantoin (300 μg) and Chloramphenicol (30 μg). The plates were incubated for 24 h at 37°C and results documented as sensitive, intermediate or resistant based on the elucidation of the diameter of the zone of inhibition.

### Multiple Antibiotic Resistance Index

The Multiple antibiotic resistance index was estimated using the formula: MARI=a/b, where a = is the number of antibiotics to which the isolates exhibited resistance and b = is the total number of antibiotics which the resistant isolates were subjected to. MARI value of more than 0.2 recommends that the isolates are from environ where there are excessive or indiscriminate use of antibiotics (Blanco et al., 2008).

### Molecular Detection of Virulence Genes

The identification of virulence genes in the *Vibrio* species was done through polymerase chain reaction (PCR) analyses. The targeted virulence genes include toxin coregulated pilus (*tcp*), toxin regulon (*toxR*), outer membrane protein (*ompU*), zonula occludens toxin (*zot*), cholera toxin gene (*ctx*) and *Vibrio* pathogenicity island (*VPI*) in *V. mimicus*. Others include *V. vulnificus* virulence genes (*vcgC* and *vcgE*) in *V. vulnificus*, extracellular haemolysin gene (*vfh*), heme utilization protein gene (*hupO*), V. fluvialis protease gene (*vfpA*) and heat stable enterotoxin (*stn*) in *V. fluvialis*. Each reaction comprises a preliminary denaturation at 94°C for 4 min, accompanied by 35 cycles each consisting of an initial denaturation at 94°C for 40 sec followed by annealing (52 °C and 62 °C) and extension steps for 90 sec while final elongation was 72°C for 6 min. Primers and their annealing temperatures are presented in **Appendix 2** (**Supplementary Material**).

### Detection of Antibiotic Resistance Genes Among the *Vibrio* Species

Also, PCR analysis was employed to identify various antibiotic resistant genes in the *Vibrio* species using pairs of specific primers and cycling conditions as follows: initial denaturation of 94°C for 5 min, accompanied by 35 cycles of 94°C for 1 min, annealing temperatures (60-62°C) for 45 s and 72°C for 91 s, final elongation step at 72°C for 5 mins. The selected targets genes were: aminoglycoside resistance genes (*strA*, *aadA*, *aacC2*, *apHAI* and *apHAII*), phenicols resistance genes (*catI, catII* and *cmlA1*), β-lactamases-encoding genes (ampC, *blaTEM* and *bla*Z) and Carbapenem resistance genes (*bla*GES*, bla*OXA*-*48*, bla*IMP*, bla*VIM*, bla*KPC, and *qnr*VC). The oligonucleotide sequences, amplicon sizes and annealing temperatures of the primers used in this study are presented in **Appendix 3** (**Supplementary Material**).

## Results

**Figure 1:**
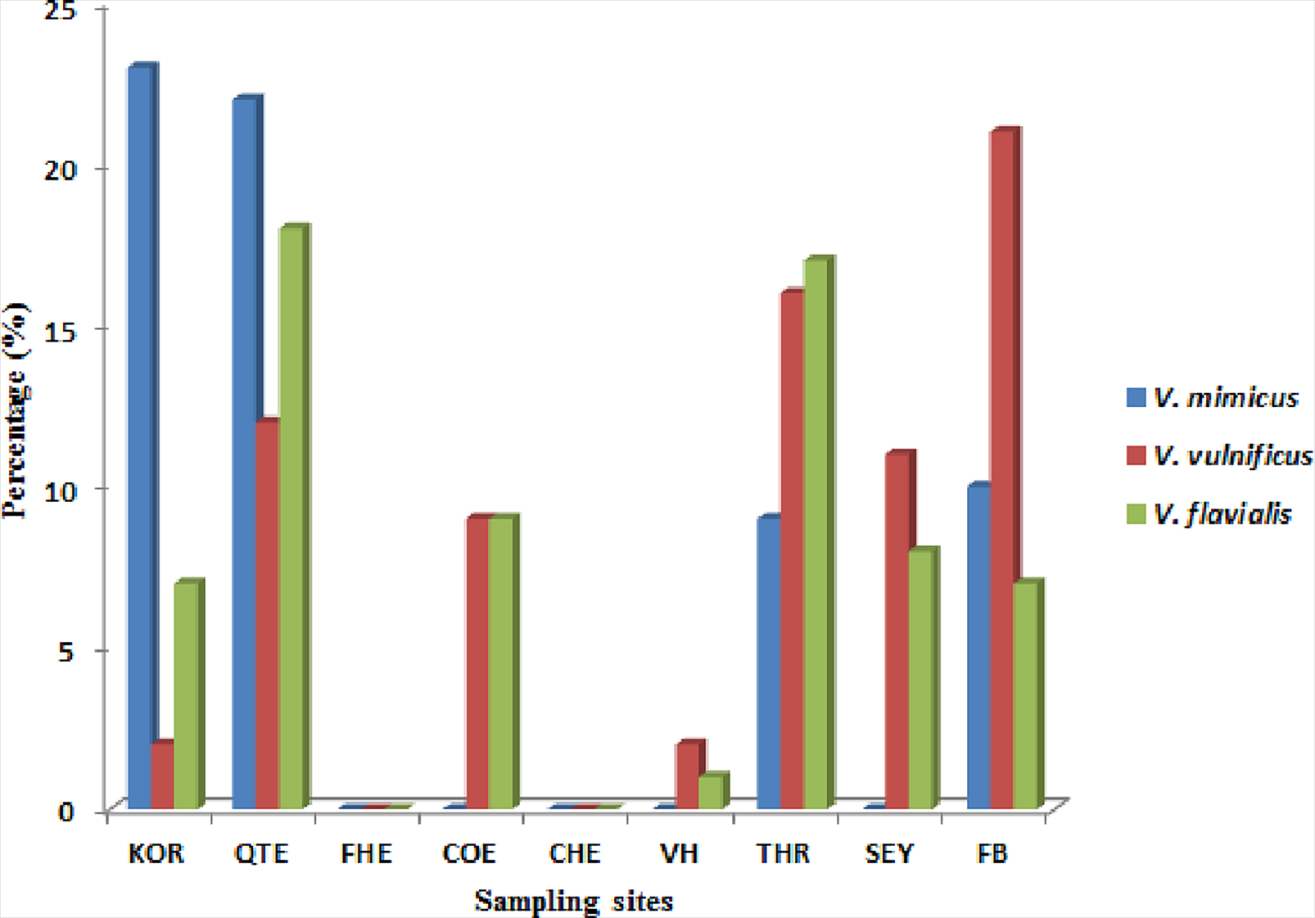
Frequency of *Vibrio* species recovered from the different site.

**Figure 2:**
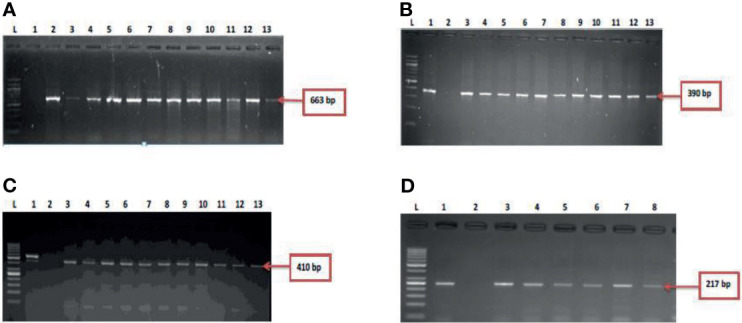
**(A)** = gel electrophoresis showing the 16s rRNA gene among isolates belonging to the *Vibrio* genus. Lane L: Molecular DNA-marker (100 bp); Lane 1: Negative control (-ve); Lane 2-13: positive isolates of 16s rRNA gene (663 bp): **(B)** = Gel electrophoresis showing the *vmh* gene among isolates belonging to the *V. mimicus*. Lane L: Molecular DNA-Marker (100 bp), lane 1: positive control (DSM 19130), Lane 2: Negative control (-ve); Lane 3-13: positive isolates of *vmh* gene (663 bp); **(C)** = Gel electrophoresis showing the *hsp60* gene among isolates belonging to the *V. vulnificus.* Lane L: Molecular DNA-marker (50 bp), lane 1: positive control (DSM 10143), Lane 2: Negative control (-ve); Lane 3-13: positive isolates of *hsp60* gene (410 bp) and **(D)** = **Figure 2D**: Gel electrophoresis showing the *ToxR* gene among isolates belonging to the *V. fluvialis.* Lane L: Molecular DNA-marker (50 bp), lane 1: positive control (DSM 19283), Lane 2: Negative control (-ve); Lane 3-8: positive isolates of *ToxR* gene (217 bp).

During the study period, a total of 695 presumptive *Vibrio* isolates were recovered from all the sampling sites. PCR analysis was used to confirm 690 isolates as *Vibrio* isolates. Of the confirmed *Vibrio* species: *V. mimicus*, *V. fluvialis* and *V. vulnificus* accounted for 40(6.7%), 41(6.8%) and 37(6.2%) respectively from seven of the nine sampling sites. Other isolates not confirmed could belong to *Vibrio* species not identified in this study. Confirmation and delineation of *Vibrio* species recovered from the different sampling sites is shown in **Table 2**. The frequency of *Vibrio* species recovered from the different site **Figure 1**, while the molecular gel electrophoresis delineating *Vibrio* into species is shown in **Figure 2**.

**Table 2:** Confirmation and delineation of *Vibrio* species recovered from the different sampling sites.

Sampling site	Presumptive *V*ibrio isolates	Confirmed isolates	*V*. *mimicus*	*V*. *vulnificus*	*V*. *fluvialis*
Komani River (KOR)	68	56	13	1	4
QT WWTP effluent (QTE)	96	82	18	10	15
Frontier hospital effluent (FHE)	103	94	0	0	0
Cofimvaba River (COE)	51	45	0	4	4
Cofimvaba Hospital effluent (CHE)	100	90	0	0	0
Victoria Hospital effluent (VH)	100	98	0	2	1
Thyume River (THR)	85	69	6	10	12
Seymour up and down stream (SEY)	54	37	0	4	3
Fort Beaufort downstream (FB)	38	29	3	6	2
**Total**	**695**	600 (86)	40 (6.7)	37 (6.2)	41 (6.8)

### Distribution of Antimicrobial Susceptibility Profile of the *Vibrio* Species

All 118 confirmed *Vibrio* species were subjected to seven antimicrobial classes comprising of twelve antibiotics. The antibiogram of the *Vibrio* isolates from all sampling sites showed varying degrees of resistance to most of the antibiotics used. A 100% resistance pattern was observed among all 118 isolates to azithromycin and polymyxin B. More than 70% of the isolates also showed resistance to ampicillin, ampicillin-sulbactam, cefuroxime and chloramphenicol. The highest susceptibility among the isolates was observed with imipenem and meropenem antibiotics. Meropenem susceptibility among *V. mimicus* and *V. fluvialis* was greater than 60%. A resistance pattern of more than 50% was observed among all the isolates to kanamycin, while fluoroquinolones had variations in the susceptibility pattern. High resistance level was observed with ofloxacin (62%) and ciprofloxacin (16%). The distribution of the antibiotic susceptibility pattern is shown in **Table 3**. The multiple antibiotic resistance patterns among the *Vibrio* species showed a range of 0.3 and 0.8. The multi-drug resistance profile among the *Vibrio* species is shown in **Table 4**.

**Table 3:** Distribution of antimicrobial susceptibility of *Vibrio* species recovered in all sampling sites.

Antimicrobial class	Antibiotics	*V. mimicus* (n = 40)			*V. vulnificus* (n = 37)			*V. fluvialis* (n = 41)		
		S	I	R	S	I	R	S	I	R
**Penicillin**	Ampicillin (10 µg)	1 (2.5)	1 (2.5)	38 (95.0)	0 (0)	0 (0)	37 (100)	1 (2.4)	4 (9.8)	36 (87.8)
**β-lactam combination agent**	Ampicillin-Sulbactam) (10/10 µg)	5 (12.5)	6 (15.0)	29 (72.5)	1 (2.7)	0 (0)	36 (97.3)	4 (9.8)	0 (0)	37 (90.2)
**Carbapenems**	Imipenem (10 µg)	21 (52.5)	17 (42.5)	2 (5.0)	1 (2.7)	10 (27.0)	26 (70.3)	17 (41.5)	13 (31.7)	11 (26.8)
	Meropenem (10 µg)	25 (62.5)	8 (20.0)	7 (17.5)	13 (35.1)	7 (18.9)	17 (45.9)	27 (65.9)	6 (14.6)	8 (19.5)
**Aminoglycosides**	Kanamycin (30 µg)	6 (15)	10 (25.0)	24 (60.0)	9 (24.3)	3 (8.1)	25 (67.8)	10 (24.4)	10 (24.4)	21 (51.2)
**Fluoroquinolones**	Ciprofloxacin (5 µg)	17 (42.5)	19 (47.5)	4 (10.0)	9 (24.3)	24 (64.9)	4 (10.8)	12 (29.3)	18 (43.9)	11 (26.8)
	Ofloxacin (5 µg)	10 (25.0)	6 (15.0)	24 (60.0)	4 (10.8)	11 (29.7)	22 (59.5)	12 (29.3)	2 (4.9)	27 (65.9)
**Cephalosporins**	Cefuroxime (30 µg)	6 (15.0)	1 (2.5)	33 (82.5)	0 (0)	2 (5.4)	35 (94.6)	1 (2.4)	2 (4.9)	28 (68.3)
**Lipopeptide**	Polymyxin B (300 µg)	0 (0)	0 (0)	40 (100)	0 (0)	0 (0)	37 (100)	0 (0)	0 (0)	41 (100)
**Macrolide**	Azithromycin (15 µg)	0 (0)	0 (0)	40 (100)	0 (0)	0 (0)	37 (100)	0 (0)	0 (0)	41 (100)
**Nitrofurans**	Nitrofurantoin (300 µg)	1 (2.5)	0 (0)	39 (97.5)	0 (0)	0 (0)	37 (100)	0 (0)	0 (0)	41 (100)
**Phenicols**	Chloramphenicol (30 µg)	0 (0)	0 (0)	40 (100)	0 (0)	0 (0)	37 (100)	0 (0)	3 (7.3)	38 (92.7)

AMP, Ampicillin; SAM, Ampicillin-sulbactam; IPM, Imipenem; MEM, Meropenem; KAN, Kanamycin; CIP, Ciprofloxacin; OFX, Ofloxacin; CXM, Cefuroxime; PB, Polymyxin B; AZM, Azithromycin; NIT, Nitrofurantoin; CHL, Chloramphenicol.

**Table 4:** Multiple antibiotic resistance pattern and MARI of *Vibrio* isolates.

Name of species	No. of isolates	Antibiotic resistance pattern	MAR1
*V. mimicus*	3	AMP-CHL-PB-KAN-AZM-CXM-SAM	0.6
	7	AMP-CHL-NIT-PB-KAN-AZM-CXM-SAM	0.7
	5	IMP-AMP-CHL-NIN-PB-KAN-AZM-CXM-0FX-SAM	0.8
	2	AMP-CHL- NIT- PB- KAN- AZM- CXM	0.6
	2	AMP-CHL-NIT-PB-KAN-MEM-AZM-CXM-OFX-SAM	0.8
	3	AMP-CHL- NIT- PB- KAN-MEM-AZM-CXM-SAM	0.8
	3	AMP-CHL-NIT-PB-AZM-CXM-OFX-SAM	0.7
	1	AMP-CHL-NIT-PB-KAN-AZM-CXM	0.6
	1	AMP-CHL-NIT-PB-AZM	0.4
	2	AMP-CHL-NIT-PB-AZM-CXM	0.5
	2	AMP-CHL-NIT-PB-KAN-MEM-AZM-SAM	0.7
	2	CHL-PB-KAN-AZM-CXM-OFX	0.6
	3	CIP-AMP-CHL-NIT-PB-KAN-AZM-CXM-OFX-SAM	0.8
	2	AMP-CHL-NIT-PB-AZM-OFX-SAM	0.6
	1	AMP-CHL-NIT-PB-MEM-AZM-CXM-OFX-SAM	0.8
	1	CIP-IMP-AMP-C-NIT-PB-AZM-CXM-OFX-SAM	0.8
	5	IMP-AMP-CHL-NIT-PB-AZM-CXM-SAM	0.7
*V. vulnificus*	1	IMP-AMP-CHL-NIT-PB-MEM-AZM-CXM-SAM	0.8
	5	IMP-AMP-CHL-NIT-PB-KAN-MEM-AZM-CXM-SAM	0.8
	5	AMP-CHL-NIT-PB-AZM-CXM-OFX-SAM	0.7
	1	IMP-AMP-CHL-NIT-PB-AZM-CXM-OFX-SAM	0.8
	6	IMP-AMP-CHL-NIT-PB-KAN-AZM-CXM-SAM	0.8
	5	IMP-AMP-CHL-NIT-PB-KAN-MEM-AZM-CXM-SAM	0.8
	1	AMP-CHL-NIT-PB-KAN-MEM-AZM-CXM-SAM	0.8
	1	AMP-CHL-NIT-PB-KAN-AZM-OFX-SAM	0.7
	2	AMP-CHL-NIT-PB-MEM-AZM-CXM-OFX-SAM	0.8
	4	CIP-AMP-CHL-NIT-PB-MEM-AZM-CXM-OFX-SAM	0.8
	1	CIP-IMP-AMP-CHL-NIT-PB-KAN-AZM-CXM-SAM	0.8
	2	NIT-PB-AZM-CXM-OFX-SAM	0.5
*V. fluvialis*	1	AMP-CHL-NIT-PB-MEM-AZM-CXM	0.6
	1	NIT-PB-MEM-AZM-CXM	0.4
	1	NIT-PB-AZM-CXM	0.3
	3	CIP-IMP-AMP-CHL-NIT-PB-AZM-CXM-SAM	0.8
	12	AMP-CHL-NIT-PB-KAN-AZM-CXM-OFX-SAM	0.8
	8	AMP-CHL-NIT-PB-AZM-CXM-OFX-SAM	0.7
	3	IMP-AMP-CHL-NIT-PB-KAN-MEM-AZM-CXM-SAM	0.8
	5	AMP-CHL-NIT-PB-AZM-CXM-OFX-SAM	0.7

### Frequency of Virulence Genes Among the *Vibrio* Species

**Figure 3:**
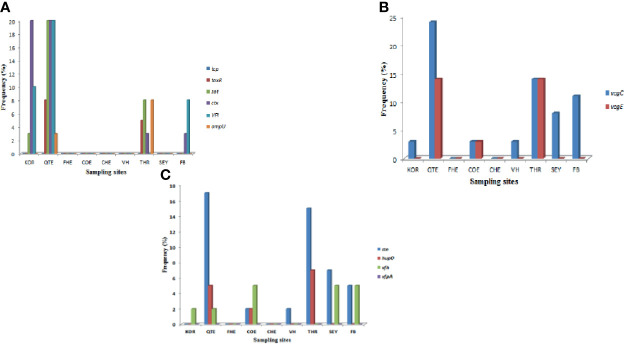
**(A)** = Frequency of *V. mimicus* virulence gene from all sampling sites, **(B)** = Frequency of *V. vulnificus* virulence genes from all sampling sites and **(C)** is Frequency of *V. fluvialis* virulence genes from all sampling sites.

**Figure 4:**
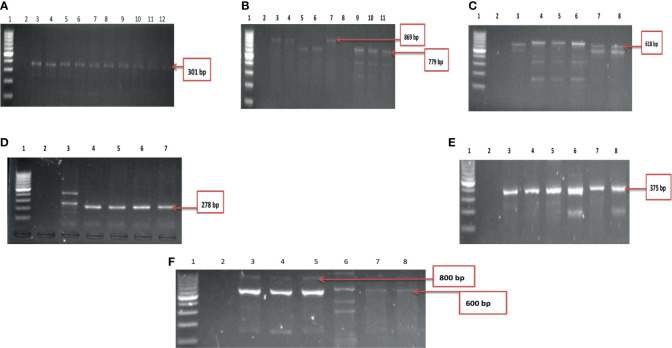
**(A)** Gel electrophoresis showing the *ctx* gene among isolates belonging to *V. mimicus.* Lane 1: Molecular Marker (100 bp), lane 2: Negative control; Lane 3-12: positive isolates of *ctx* gene (301 bp). **(B)** Gel multiplex electrophoresis showing the *ompU and toxR* genes among isolates belonging to *V. mimicus.* Lane 1: Molecular Marker (100 bp), lane 2: Negative control; Lane 3, 4, 7 and 8: positive isolates of *ompU* gene (869 bp). Lane 5, 6, 9-11: positive isolates of *toxR* gene (779 bp). **(C)** Gel electrophoresis showing the *VPI* gene among isolates belonging to *V. mimicus.* Lane 1: Molecular Marker (100 bp), lane 2: Negative control; Lane 3-8: positive isolates of *VPI gene* (618 bp). **(D)** Gel electrophoresis showing the *vcgC* and *vcgE* gene among isolates belonging to *V. vulnificus.* Lane 1: Molecular Marker (100 bp), lane 2: Negative control; Lane 3-7: positive isolates of *vcgC* and *vcgE* gene (278 bp). **(E)** Gel electrophoresis showing the *stn* gene among isolates belonging to *V. fluvialis.* Lane 1: Molecular Marker (100 bp), lane 2: Negative control; Lane 3-8: positive isolates of *stn* gene (375 bp). **(F)** Gel multiplex electrophoresis showing the *vfh and hupO* gene among isolates belonging to *V. fluvialis.* Lane 1: Molecular Marker (100 bp), lane 2: Negative control; Lane 3-7: positive isolates of *vfh* gene (800 bp). Lane 8: positive isolates of *hupO* gene (600 bp).

A total of ten of the twelve virulence genes were detected among the three *Vibrio* species recovered from all the sites. *Vibrio mimicus* harboured five virulence genes in the following proportion: *toxR* (12.5%), *zot* (32.5%), *ctx* (45%), *VPI* (37.5%), *tcp* (0), and *ompU* (10%). Two virulence genes detected among the *V. vulnificus* isolates were *vcgC* (63.1%) and *vcgE* (29%). Three of the four virulence genes were likewise detected among the *V. fluvialis* isolates recovered from all the sampling sites in the following proportion: *stn* (48.8%), *hupO* (14.6%), *vfpA* (0) and *vfh* (19.5%). **Figures 3A–C** represent the frequency of virulence genes among the three *Vibrio* species (*V. mimicus*, *V. vulnificus* and *V. fluvialis*) recovered from all sampling sites. The gel representative pictures for the amplification of the virulence genes for the three *Vibrio* species are shown in **Figure 4**.

### Detection of Resistance Genes Among the *Vibrio* Species

**Figure 5:**
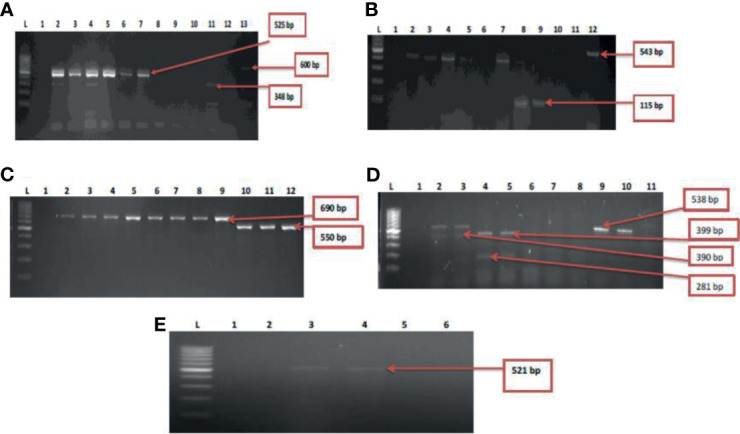
**(A)** = Gel multiplex electrophoresis showing the *aphA1, aadA and strA* genes among the *Vibrio species.* Lane L: Molecular Marker (100 bp), lane 1: Negative control; Lane 2-7: positive isolates of *aadA* gene (525 bp). Lane 11: positive isolate of *strA* gene (348 bp) and lane 13: positive isolate of *aphA1* gene (600 bp), **(B)** = Gel multiplex electrophoresis showing the *cmlA1 and catII* genes among the *Vibrio species.* Lane L: Molecular Marker (100 bp), lane 1: Negative control; Lane 2-5, 7, 10 and 12: positive isolates of *catII* gene (543 bp). Lane 8 and 9: positive isolate of *cm/A/*gene (115 bp), **(C)** = Gel multiplex electrophoresis showing the *blaTEM and* ampC genes among the *Vibrio species.* Lane L: Molecular Marker (100 bp), lane 1: Negative control; Lane 2-9: positive isolates of *bla*TEM gene (690 bp). Lane 10-12: positive isolate of ampC gene (550 bp), **(D)** = Gel multiplex electrophoresis showing the *bla*KPC*, bla*GES*, bla*VIM and *bla*OXA*-*48 genes among the *Vibrio species.* Lane L: Molecular DNA-Marker (100 bp), lane 1: Negative control; Lane 2, 3, 9 and 10: positive isolates of *bla*KPC gene (543 bp). Lane 4 and 5: positive isolate of *bla*GES gene (399 bp). Lane 2 and 3: positive *bla*VIM isolates (390 bp). Lane 4 and 5: positive isolates of *bla*OXA-48 (281 bp), **(E)** = Gel electrophoresis showing the *qnr*VC genes among the *Vibrio species.* Lane L: Molecular DNA-marker (100 bp), lane 1: Negative control; Lane 3 and 4: positive isolates of *qnr*VC gene (521 bp).

Five resistant genes encoding for aminoglycoside were examined in this study and three were detected of which *aadA* (50%) was observed as the most prevalent while *strA* was the least with a prevalence of 4.3%. *V. mimicus* was devoid of any *strA* gene. However, 26% of *V. mimicus* solely harboured *aph(3)- Ia (aphA1*)a gene among all the *Vibrio* isolates. For the phenicols, three resistance genes were detected in 5.2% among the confirmed *Vibrio* species. 44.3% of the *Vibrio* species harboured the *catII* gene while *catI* gene was not detected among the three *Vibrio* species studied. The ampC gene was identified in 42% of the *Vibrio* isolates, *bla*TEM in 53.3% of the *Vibrio* isolates, and no isolate harboured the *bla*Z gene. The frequency of resistance genes among the carbapenems class of antibiotics detected in the studied isolates varied from 5.6% for and 29.6%. The frequency of resistance genes with carbapenem was *bla*GES (16.6%), *bla*OXA-48 (11.3%), *bla*IMP (15%), *bla*VIM (29.6%) and *bla*KPC (5.6%). Among the resistance genes to fluoroquinolones assayed, only two isolates representing 2.8% harboured the *qnr*VC gene in all the isolates studied. The distribution of the resistance genes among the *Vibrio* resistance isolates is shown in **Table 5**. The gel representative pictures for the amplification of the resistance genes for aminoglycoside, phenicols, beta-lactams, carbapenems and fluoroquinolones are shown in **Figure 5** respectively.

**Table 5:** Distribution of antimicrobial resistant genes among *Vibrio* resistant species.

Antimicrobial family	Total number of *Vibrio* species resistant to antibiotics	Resistant genes	*Vibrio* species			Total percentage of resistant genes
			*V. mimicus*	*V. vulnificus*	*V. fluvialis*	
Aminoglycosides	70	*strA*	–	2	1	4.3
		*aadA*	6	12	17	50.0
		*aac(3*)- IIa (*aacC2*) a	–	–	–	–
		*aph*(3)- Ia (*aphA*1) a	18	–	–	26.0
		*aph*(3)- IIa (*aphA2*) a	–	–	–	–
Phenicols	115	*cmlA1*	2	4	–	5.2
		*catI*	–	–	–	–
		*catII*	18	16	17	44.3
Beta-lactams	102	*ampC*	26	16	5	42.0
		*blaTEM*	38	15	35	53.3
		*blaZ*	–	–	–	–
Carbapenems	71	*GES*	4	7	1	16.6
		*bla*OXA-48	3	3	2	11.3
		*blaI*MP	2	4	5	15.5
		*bla*VIM	1	15	5	29.6
		*bla*KPC	–	4	–	5.6
Fluoroquinolones	92	*qnrVC*	–	–	2	2.8
				

## Discussion

Water is considered as one of the basic necessities for the existence of all living organisms. South Africa is a semi-arid region which obtains minimal amount of rain annually. In lieu of this and with the incessant shortages of portable and clean water in the country, most inhabitants, especially in the rural areas have resorted to different alternatives for water sources such as river and dams for their routine domestic activities (Meissner et al., 2018). Additionally, the upsurge in various informal settlements around the country as well as the present poor conditions of some wastewater treatment plants have significantly contributed to the diminished quality of water sources found in most of the rural areas (Govender et al., 2011). Most hospital settings in the rural areas do not treat their effluents and are channelled with municipal wastewater prior (Edokpayi et al., 2017). Often times, compromised sewage pipelines leak their contents into the environment which eventually end up in adjourning streams and rivers.

Our study detected three *Vibrio* species (*V. mimicus*, *V. fluvialis* and *V. vulnificus*) from all sampling sites through molecular analyses. While culture-based techniques are relied upon for detecting microbial presence in various environments, however current molecular methods are comparatively more specific and sensitive than culture-based methods (Franco-Duarte et al., 2019). Specifically, the confirmation of *Vibrio* isolates from targeted sites in this study was achieved by molecular amplification of 16SrRNA gene followed by target genes *hsp60*, *toxR* and *vmh* for *V*. *vulnificus*, *V. fluvialis* and *V. mimicus* isolates. Previous studies have also employed similar molecular methods in the confirmation and delineation of *Vibrio* isolates into species (Kim et al., 2015; Okoh et al., 2015; Guardioala-Avila et al., 2016). Overall, *Vibrio* species recovered during this study were absent in two sampling sites (FHE and CHE) and could be *Vibrio* isolates belonging to other species not identified in this study. The presence of the *Vibrio* isolates from seven sampling sites is shown in **Table 5**. The QTE site had the highest recovery of the *Vibrio* isolates and poor treatment plant performance could have contributed to the recovery of the *Vibrio* isolates in the final effluent sample. The occurrence of pathogens in waterbodies is often due to the aftereffect from faecal contamination involving humans and animals (Penakalapati et al., 2017). The uncontrolled discharge of sewage as well as nonchalant management of wastewater treatment plants have been incriminated in water pollution of microbial origin especially around the rural settlements in South Africa (Teklehaimanot et al., 2015). The antibiotic sensitivity patterns among the *Vibrio* isolates recorded showed varying degrees of resistance pattern. All *Vibrio* species in the study exhibited more than 95% resistance to polymyxin B, chloramphenicol and nitrofurantoin. Results from our findings on the resistance pattern among the *Vibrio* isolates were slightly higher to those reported by Beshiru et al. (2020). In general, all confirmed *Vibrio* species from our study showed more than 85% resistance pattern to ampicillin. The carbapenems have been proven to be broad spectrum antibiotic and active against a diverse range of Gram positive and Gram negative bacteria (Papp-Wallace et al., 2011). From our study, a high susceptibility pattern was observed with the carbapenems (imipenem and meropenem) against *V. mimicus* and *V. fluvialis*. However, low sensitivity pattern was noticed among *V*. *vulnificus* isolates. Kanamycin also exhibited a sensitivity pattern of more than 50% with the test isolates and in strong agreement with previous report by Maje et al. (2020). A third generation antibiotic (cefuroxime) also showed a resistance pattern of more than 75% among the *Vibrio* isolates and is comparable with the findings by Manjusha and Sarita (2011). Ofloxacin also had a higher sensitivity pattern compared to ciprofloxacin but however, more isolates had an intermediate pattern of sensitivity with ciprofloxacin.

Multiple antibiotic resistance (MAR) analysis has been introduced to discern bacteria from sources using antimicrobial agents regularly approved for human therapy. In bacteria, MAR is most frequently associated with the presence of plasmids which harbours single or multiple resistance genes (Ruppé et al., 2015). Multiple antibiotic resistance patterns among the *Vibrio* species had a range of 0.5 to 0.8 while most of the isolates had a MARI of 0.8. This inferred that the *Vibrio* isolates originated from sources where antibiotic were greatly utilized.

Microbial virulence factors involve a wide range of molecules produced by pathogenic bacteria with increasing propensity to elude their host defence and cause infection. Also, some secretes microbial products which have the ability to gain entry into the host cell and utilize their mechanism assisting in the attainment of infection (Kitamoto et al., 2016). Virulence genes in *Vibrio* species have assisted in the initiation of infection ranging from diarrhoea, gastroenteritis, cholera, wound and blood infections (Schirmeister et al., 2014). It is commonly assumed that environmental bacterial strains are devoid of virulence traits that are present in clinical strains. Nonetheless, recent studies have reported that virulence genes, or their homologues could be found in strains from environmental sources and that acquiring such genes may have occurred in the aquatic environment (Persson et al., 2009). From this study, ten virulence genes were detected in varying frequencies among the *Vibrio* species isolated. The *toxR* virulence is made up of a set of genes encoding proteins that encourages internal colonization, toxin production and survival in the host cell coordinately expressed and dependent upon transcriptional activator (*toxR*) (Bina et al., 2003). The *toxR* gene was detected among *V. mimicus* isolates from two sampling sites (QTE and THR). This study affirms with previous report (Chen et al., 2011) detecting *toxR* gene in *V.mimicus* from sea foods. *Zot* gene was initially detected in *Vibrio cholerae* and encoded by ctx prophage while the core part of *ctx* gene comprises of many structural genes including zot genes which has significance in phage morphogenesis and assembly (Pant et al., 2020). The detection of *ctx* and *zot* genes from our study is comparable to the reports by Menezes et al. (2014). The presence of a *zot* gene in *ctx-*positive *V. mimicus* indicates a possible significant role of *zot* in the toxigenicity of the species. *Vibrio* pathogenicity island (*VPI*) gene and *ompU* (encoding a porin in the outer membrane of the bacteria) were also detected among some *V. mimicus* isolates. *VPI* has been attributed to be an essential virulence gene cluster for colonization of the human intestine as well as receptor for infection in most toxigenic *Vibrio* strains (Labbate et al., 2016). Detection of *VPI* and *ompU* genes among some *V. mimicus* in this study is in agreement with previous study by Guardiola-Avila et al. (2020). The detection of virulence correlated gene in this study among the *V. vulnificus* isolates can be distinguished apparently as clinical (*vcgE*) and environmental (*vcgC*) (Bier et al., 2015). Our finding is in accord with the report by Warner and Oliver (2008) who detected *vcgE* (47%) and *vcgC* (53%) from oysters’ isolates and water surrounding oysters harvest in North Carolina. Among the *V. fluvialis* isolates, three genes detected were *stn* (48%), *hupO* (14.6%) and *vfh* (19.5%). These factors have been reported to assist in haemolysin production which intensifies the pathogenic prowess of *V. fluvialis* and increase diarrhoea in infected patients (Liang et al., 2013). A similar trend was documented by Fri et al. (2017) that also identified similar virulence genes from a dusky farm and estuary in South Africa.

Antibiotic resistance is one of the imminent challenges to global health and with the current trends in escalating environmental contaminants such as antibiotic resistance genes; natural environments likely to be potential repositories for the spread of antibiotic resistance (Hernando-Amado et al., 2020). Aminoglycosides are potent antibiotics that function *via* the inhibition of protein synthesis (Wasserman et al., 2015). We found in this study that there was a wide range from relatively moderate to low and eventually no detection of aminoglycoside resistance genes among the *Vibrio* isolates. The detection of streptomycin resistance genes (*aadA*, *strA*) and gentamicin resistance genes (*aphA2*) from our study varied among the *Vibrio* species which corresponds with a previous study by Adefisoye and Okoh (2017). Chloramphenicol resistance genes was analysed among all *Vibrio* species showing phenotypic resistance to the antibiotic. Chloramphenicol resistance is attributed to the enzymatic inhibition of the drug facilitated by chloramphenicol acetyl-transferases (CAT) (Alcala et al., 2020). The detection of *cat* resistance genes among some *Vibrio* isolates from our study is akin with other studies (Yoo et al., 2003; Zhang et al., 2019; Håkonsholm et al., 2020). Beta lactams are the most broadly used class of antimicrobial agents, characterized by minimal toxicity and employed in the treatment of various bacterial ailments including those attributed with different *Vibrio* species (Bush and Bradford, 2016). Apparently, resistance among the beta-lactam drugs continues to be on the surge. The broad spectrum TEM-beta lactamase has been reported to be widely distributed in plasmids (Tooke et al., 2019). AmpC genes are situated chromosomally but nonetheless, plasmid associated variants among some bacteria have become increasingly recognized and disseminated *via* horizontal transfer (Lan et al., 2017). Our study detected the presence of ampC and *bla*TEM genes among the *Vibrio* species. Carbapenems introduction as broader antimicrobial spectrum are usually the last resort for most antimicrobial agents due to the proliferation of resistance among most antimicrobial classes. But resistance has been observed among the carbapenems. Our study detected five beta lactamase resistance genes to carbapenems. Detection of resistance genes from this study correlates with recent report (Elbadawi et al., 2021) identifying carbapenem resistance genes (*bla*OXA*-*48*, bla*IMP*, bla*VIM and *bla*KPC) among Gram negative isolates in Sudan. In addition, *bla*GES was identified among the three *Vibrio* species in our study. Documented reports have shown limited *Vibrio* carrying *bla*GES genes (Dahanayake et al., 2020; Hossain et al., 2020). These beta-lactam resistance genes are frequently identified in *Enterobacteriaceae* which portends concern on the impact of sewage in aquatic environment, giving rise to drug resistance bacteria and fortified selective pressure increasing the advent and dissemination of antimicrobial resistance (Fresia et al., 2019) The acquisition of these genes in *Vibrio* isolates in our study may have been through plasmid mediation in the aquatic environment from other bacteria strains. The addition of fluoride to quinolone creates fluoroquinolones with enhanced bactericidal activity. However, the plasmid- mediated quinolone resistance gene (*qnrVC*) was identified in two *V. fluvialis* isolates from our study and similar trend have been reported by Vinothkumar et al. (2016).

## Conclusion

Our present study is a broad assessment of the antimicrobial susceptibility of various *Vibrio* species that were successfully recovered from aquatic and hospital environments. Detection of multiple drug resistance *Vibrio* species, isolates harbouring virulence genes as well as the detection of antimicrobial resistance genes suggests risk for public health concern. The maintenance of dysfunctional wastewater treatment plants as well as burst sewage pipes in rural settlements should be rectified to further prevent the leakages of sewage into the surrounding environments. More importantly, the promotion of proper antibiotic stewardships should be upheld in the society to forestall the continuous propagation and sustenance of antimicrobial resistance among bacteria population in nature.

## Data Availability Statement

The raw data supporting the conclusions of this article will be made available by the authors, without undue reservation.

## Author Contributions

UN, Conceptualization. OG and BI, methodology. OG and TD, formal analyses. NU, resources. OG and TD, writing—original draft preparation. OG, TD, BI, OO, AO, and UN, writing—review and editing. UN, supervision. All authors contributed to the article and approved the submitted version.

## Conflict of Interest

The authors declare that the research was conducted in the absence of any commercial or financial relationships that could be construed as a potential conflict of interest.

## Publisher’s Note

All claims expressed in this article are solely those of the authors and do not necessarily represent those of their affiliated organizations, or those of the publisher, the editors and the reviewers. Any product that may be evaluated in this article, or claim that may be made by its manufacturer, is not guaranteed or endorsed by the publisher.
