# Supplementary material for: Virulence and Antibiotic Resistance Characteristics of Vibrio Isolates From Rustic Environmental Freshwaters
Source: Front Cell Infect Microbiol. 2021 Aug 19;11:732001. doi: 10.3389/fcimb.2021.732001 (PMC8416912; doi:10.3389/fcimb.2021.732001)
Supplement: Supplementary file 1 [file DataSheet_1.docx]

References

Baddour, M.M., AbuElKheir, M.M. and Fatani, A.J., 2007. Comparison of *mecA* polymerase chain reaction with phenotypic methods for the detection of methicillin-resistant *Staphylococcus aureus*. *Cur. Microbiol*. 55(6), pp.473-479.

Bailey, J., Pinyon, J., Anantham, S. and Hall, R., 2010. Commensal Escherichia coli of healthy humans: a reservoir for antibiotic-resistance determinants. *J. Med. Microbiol*., 59(11), pp.1331-1339.

Bi, K., Miyoshi, S.I., Tomochika, K.I. and Shinoda, S., 2001. Detection of virulence associated genes in clinical strains of *Vibrio mimicus*. *Microbiol. Immunol*. 45(8), pp.613-616.

Blasco, M.D., Esteve, C. and Alcaide, E., 2008. Multiresistant waterborne pathogens isolated from water reservoirs and cooling systems. *J. Appl. Microbiol*. 105(2), pp.469-475.

Chakraborty, R., Sinha, S., Mukhopadhyay, A.K., Asakura, M., Yamasaki, S., Bhattacharya, S.K., Nair, G., Ramamurthy, T. (2006) Species-specific identification of *Vibrio fluvialis* by PCR targeted to the conserved transcriptional activation and variable membrane tether regions of the toxR gene. *J. Med. Microbiol*. 55, 805e808. doi:10.1099/jmm.0.46395-0.

Dallenne, C., Da Costa, A., Decré, D., Favier, C. and Arlet, G., 2010. Development of a set of multiplex PCR assays for the detection of genes encoding important β-lactamases in Enterobacteriaceae. *J. Antimicrob. Chemother*. 65(3), pp.490-495.

Kwok, A.Y., Wilson, J.T., Coulthart, M., Ng, L.K., Mutharia, L. and Chow, A.W., 2002. Phylogenetic study and identification of human pathogenic *Vibrio* species based on partial hsp 60 gene sequences. *Canad. J. Microbiol*, 48(10), pp.903-910.

Mantri, C.K., Mohapatra, S.S., Ramamurthy, T., Ghosh, R., Colwell, R.R. and Singh, D.V., 2006. Septaplex PCR assay for rapid identification of *Vibrio cholerae* including detection of virulence and int SXT genes. *FEMS Microbiol. Letters*. 265(2), pp.208-214.

Maynard, C., Bekal, S., Sanschagrin, F., Levesque, R.C., Brousseau, R., Masson, L., Larivière, S. and Harel, J., 2004. Heterogeneity among virulence and antimicrobial resistance gene profiles of extraintestinal Escherichia coli isolates of animal and human origin. *J. clin. Microbiol*, 42(12), pp.5444-5452.

Post, V. and Hall, R.M., 2009. AbaR5, a large multiple-antibiotic resistance region found in *Acinetobacter baumannii*. *Antimicrob. Chemother*. 53(6), pp.2667-2671.

Rosche, T.M., Yano, Y. and Oliver, J.D., 2005. A rapid and simple PCR analysis indicates there are two subgroups of *Vibrio vulnificus* which correlate with clinical or environmental isolation. *Microbiol. Immunol*. 49(4), pp.381-389.

Shinoda, S., Nakagawa, T., Shi, L., Bi, K., Kanoh, Y., Tomochika, K.I., Miyoshi, S.I. and Shimada, T., 2004. Distribution of virulence‐associated genes in *Vibrio mimicus* isolates from clinical and environmental origins. *Microbiol. Immunol*. 48(7), pp.547-551. doi: https://doi.org/10.1111/j.1348-0421.2004.tb03551.x.

Srinivasan, V., Gillespie, B.E., Lewis, M.J., Nguyen, L.T., Headrick, S.I., Schukken, Y.H. and Oliver, S.P., 2007. Phenotypic and genotypic antimicrobial resistance patterns of *Escherichia coli* isolated from dairy cows with mastitis. *Vet. Microbiol*. 124(3-4), pp.319-328.

Wong, R.S. and Chow, A.W., 2002. Identification of enteric pathogens by heat shock protein 60 kDa (HSP60) gene sequences. *FEMS Microbiol. Letters*. 206(1), pp.107-113.

Xia, L.N., Li, L., Wu, C.M., Liu, Y.Q., Tao, X.Q., Dai, L., Qi, Y.H., Lu, L.M. and Shen, J.Z., 2010. A survey of plasmid-mediated fluoroquinolone resistance genes from Escherichia coli isolates and their dissemination in Shandong, China. *Foodborne pathog. Dis*. 7(2), pp.207-215.

Xie, Z.Y., Hu, C.Q., Chen, C., Zhang, L.P. and Ren, C.H., 2005. Investigation of seven Vibrio virulence genes among *Vibrio alginolyticus* and *Vibrio parahaemolyticus* strains from the coastal mariculture systems in Guangdong, China. *Letters Appl. Microbiol.*, *41*(2), pp.202-207.
