# Supplementary material for: Virulence and Antibiotic Resistance Characteristics of Vibrio Isolates From Rustic Environmental Freshwaters
Source: Front Cell Infect Microbiol. 2021 Aug 19;11:732001. doi: 10.3389/fcimb.2021.732001 (PMC8416912; doi:10.3389/fcimb.2021.732001)
Supplement: Supplementary file 2 [file Table_1.docx]

Appendix 1

Oligonucleotide primers for the confirmation of *Vibrio* genus and species

| **Target species** | **Target gene** | **Oligonucleotide sequence(5’----3’)** | **Amplicon size (bp)** | **Cycling conditions** | **References** |
| --- | --- | --- | --- | --- | --- |
| *Vibrio* genus | 16S rRNA | F: CGG TGAAATGCGTAGAGAT  R: TACTAGCGATTCCGAGTTC | 663 | Initial denaturation at 93°C for 15 min followed by 35 cycles of denaturation: 92°C for 40 s, annealing: 57°C for 1 min, elongation 72°C for 1.5 min, and final extension at 72°C for 7 min | Kwok *et al*. (2002) |
| *V. vulnificus* | *hsp60* | F: GTCTTAAAGCGGTTGCTGC  R: CGCTTCAAGTGCTGGTAGAAG | 410 | Initial denaturation at 94°C for 5 min, followed by 35 cycles of 94 °C  for 30 s, 55 °C for 30 s, 72°C for 30 s, and final extension  at 72°C for 10 min | Wong and Chow (2002) |
| *V. fluvialis* | *toxR* | F: GACCAG GGCTTTGAGGTGGAC  R: GGATACGGCACTTGAGTAAGACTC | 217 | Initial denaturation at 94°C for 5 min, followed by 30 cycles of 94 °C for 40 s, 65°C for 40 s, 72°C for 1 min, and final extension at 72°C for 7 min | Chakraborty et al., (2006) |
| *V. mimicus* | *vmh* | F: GGTAGCCATCAGTCTTATCACG  R:ATCGTGTCCCAATACTTCACCG | 390 | Initial denaturation at 95°C for 5 min; 35 cycles at 95 °C for 30 s, 53°C for 30 s, and 72°C for 1 min, and final extension at 72°C for 5 min | Guardiola et al., 2016 |
