# Supplementary material for: Virulence and Antibiotic Resistance Characteristics of Vibrio Isolates From Rustic Environmental Freshwaters
Source: Front Cell Infect Microbiol. 2021 Aug 19;11:732001. doi: 10.3389/fcimb.2021.732001 (PMC8416912; doi:10.3389/fcimb.2021.732001)
Supplement: Supplementary file 3 [file Table_2.docx]

Appendix 2

Primer sequences for the detection of virulence genes among the *Vibrio* species

| **Species** | **Gene** | **Oligonucleotide sequence 5’- 3’** | **Amplicon size (bp)** | **Annealing temperatures** | **References** |
| --- | --- | --- | --- | --- | --- |
| *V. mimicus* | *tcp* | F:GAAGAAGTTTRTAAAAGAAGAACA  R:GAAAGGACCTTCTTTCACGTTG | 451 | 55 ^o^C | Shinoda *et al*., 2004 |
|  | *toxR* | F:ATGTTCGGATTAGGACAC  R:TACTCACACACTTTGATGGC | 779 | 60 ^o^C | Mantri *et al*., 2006 |
|  | *ompU* | F:ACGCTGACGGAATCAACCAAAG  R:GCGGAAGTTTGGCTTGAAGTAG | 869 | 62 ^o^C | Singh *et al*., 2002 |
|  | *zot* | F:TCGCTTAACGATGGCGCGTTTT  R:AACCCCGTTTCACTTCTACCCA | 947 | 62 ^o^C | Singh *et al*., 2002 |
|  | *ctx* | F:CTCAGACGGGATTTGTTAGGCACG  R:TCTATCTCTGTAGCCCCTATTACG | 301 | 55 ^o^C | Bi *et al*., 2001 |
|  | *VPI* | F:GCAATTTAGGGGCGCGACGT  R:CCGCTCTTTCTTGATCTGGTAG | 618 | 52 ^o^C | Xie et al., 2005 |
| *V. vulnificus* | *vcgC* | F:AGCTGCCGATAGCGATCT  R:CGCTTAGGATGATCGGTG | 278 | 56 ^o^C | Rosche *et al*., 2005 |
|  | *vcgE* | F:CTCAATTGACAATGATCT  R: CGCTTAGGATGATCGGTG | 278 | 56 ^o^C | Rosche *et al*., 2005 |
| *V. fluvialis* | *vfh* | F:GCGCGTCAGTGGTGGTGAAG  R:TCGGTCGAACCGCTCTCGCTT | 800 | 61 ^o^C | Liang *et al*., 2013 |
|  | *hupO* | F:ATTACGCACAACGAGTCGAAC  R:ATTGAGATGGTAAACAGCGCC | 600 | 56 ^o^C | Liang *et al*., 2013 |
|  | *vfpA* | F:TACAACGTCAAGTTAAAGGC  R:GTAGGCGCTGTAGCCTTTCA | 1790 | 55 ^o^C | Liang *et al*., 2013 |
|  | *stn* | F:GGTGCAACATAATAAACAGTCAACAA  R:TAGTGGTATGCGTTGCCAGC | 375 | 53 ^o^C | Liang *et al*., 2013 |
