# Supplementary material for: Virulence and Antibiotic Resistance Characteristics of Vibrio Isolates From Rustic Environmental Freshwaters
Source: Front Cell Infect Microbiol. 2021 Aug 19;11:732001. doi: 10.3389/fcimb.2021.732001 (PMC8416912; doi:10.3389/fcimb.2021.732001)
Supplement: Supplementary file 4 [file Table_3.docx]

Appendix 3: Oligonucleotide primers used in the detection of antibiotic resistance genes among the *Vibrio* isolates

| Antimicrobial class | Primer | Primer sequence (5ʹ–3ʹ) | Amplicon size (bp) | Annealing temperatures | References |
| --- | --- | --- | --- | --- | --- |
| Aminoglycosides | *strA* | F: CTTGGTGATAACGGCAATTC  R: CCAATCGCAGATAGAAGGC | 384 | 50 ^o^C | Srinivasan et al., 2007 |
|  | *aadA* | F: GTGGATGGCGGCCTGAAGCC  R: AATGCCCAGTCGGCAGCG | 525 | 50 ^o^C | Srinivasan et al., 2007 |
|  | *aac(*3*)- IIa*  (*aac*C2) a | F:CGGAAGGCAATAACGGAG  R: TCGAACAGGTAGCACTGAG | 428 | 50 ^o^C | Maynard et al. (2004) |
|  | *aph*(3)- *Ia* (*aphA1) a* | F:ATGGGCTCGCGATAATGTC  R: CTCACCGAGGCAGTTCCAT | 600 | 50 ^o^C | Maynard et al. (2004) |
|  | *aph(3)*- *IIa* (*aphA2) a* | F: GAACAAGATGGATTGCACGC  R: GCTCTTCAGCAATATCACGG | 510 | 50 ^o^C | Maynard et al. (2004) |
| Phenicols | *cmlA1* | F: CACCAATCATGACCAAG  R:GGCATCACTCGGCATGGACATG | 115 | 50 ^o^C | Post and Hall (2009) |
|  | *catI* | F:AGTTGCTCAATGTACCTATAACC  R:TTGTAATTCATTAAGCATTCTGCC | 320 | 50 ^o^C | Maynard et al. (2004) |
|  | *catII* | F: ACACTTTGCCCTTTATCGTC  R: TGAAAGCCATCACATACTGC | 543 | 50 ^o^C | Maynard et al. (2004) |
| Beta-lactams | *ampC* | F: TTCTATCAAMACTGGCARCC  R: CCYTTTTATGTACCCAYGA | 550 | 50 ^o^C | Srinivasan et al., 2007 |
|  | *blaTEM* | F: TTTCGTGTCGCCCTTATTCC  R: CCGGCTCCAGATTTATCAGC | 690 | 60 ^o^C | Bailey et al. (2010) |
|  | *blaZ* | F: ACTTCAACACCTGCTTTC  R: TGACCACTTTTATCAGCAACC | 490 | 60 ^o^C | Baddour et al. (2007) |
| Carbapenems | *bla*GES | F:AGTCGGCTAGACCGGAAAG  R:TTTGTCCGTGCTCAGGAT | 399 | 57 ^o^C | Dallenne *et al*., 2010 |
|  | *bla*OXA*-48* | F:GCTTGATCGCCCTCGATT  R:GATTTGCTCCGTGGCCGAAA | 281 | 57^o^C | Dallenne *et al*., 2010 |
|  | *bla*IMP | F:TTGACACTCCATTTACDG  R:GATYGAGAATTAAGCCACYCT | 139 | 55 ^o^C | Dallenne *et al*., 2010 |
|  | *bla*VIM | F:GATGGTGTTTGGTCGCATA  R:CGAATGCGCAGCACCAG | 390 | 55 ^o^C | Dallenne *et al*., 2010 |
|  | *bla*KPC | F:CATTCAAGGGCTTTCTTGCTGC  R:ACGACGGCATAGTCATTTGC | 538 | 55 ^o^C | Dallenne *et al*., 2010 |
| fluoroquinolones | *qnrVC* | F:CCCTCGAGCATGGATAAAACAGACCAGTTATA R:CGGGATCCTTAGTCAGGAACTACTATTAAACCT | 521 | 62 ^o^C | Xia *et al*., 2010 |
